# Myotonic Dystrophy and Anesthetic Challenges: A Case Report and Review

**DOI:** 10.1155/2019/4282305

**Published:** 2019-03-20

**Authors:** Chanchal Mangla, Kimmy Bais, Joel Yarmush

**Affiliations:** Department of Anesthesiology, New York Presbyterian Brooklyn Methodist Hospital, Brooklyn, New York, USA

## Abstract

Providing anesthesia to patients with myotonic dystrophy (DM) can be very challenging due to the multisystemic effects of the disease and extreme sensitivity of these patients to sedatives, opioids, and anesthetic agents. Other factors such as hypothermia, shivering, or mechanical or electric stimulation during surgery can precipitate myotonia which is difficult to abolish and can lead to further complications. Generally, local or regional anesthesia is preferred to avoid the complications associated with general anesthesia in this group. However there are several case reports of successful use of general anesthesia (with or without volatile agents and with or without opioids). These general anesthetic cases led to postoperative admission to the regular floor or ICU. We present a case of a woman with a history of DM who underwent robotic assisted laparoscopic hysterectomy under general anesthesia and was discharged home on the same day.

## 1. Introduction

Myotonic dystrophy (DM) is the most common and severe form of the myotonic syndromes with an incidence of 1 in 8,000 newborns and prevalence of 2-14 per 100,000 population [1–3]. First described by Steinert in 1909 [1, 2], it primarily affects muscles. DM involves myotonia which is characterized by persistent muscle contractions after termination of voluntary muscle action. There are two types of DM. Type 1, known as Steinert's disease, is more common and involves distal muscle groups while type 2 involves proximal muscles. Type 1 is more closely associated with postoperative respiratory and other systemic complications [4].

## 2. Case Description

A 46-year-old African American female with a history of DM and leiomyoma of the uterus was admitted for robotic assisted laparoscopic total abdominal hysterectomy and bilateral salpingo-oophorectomy. Her DM was diagnosed during adolescence and was confirmed by genetic testing. Her symptoms were characterized by frequent myotonia and intermittent weakness of the distal extremities. Her weakness had extended to her facial and pharyngeal muscles. Family history was notable for her daughter, who was also diagnosed and treated for DM. Patient other comorbidities included bronchial asthma, chronic sinus bradycardia, cataracts, depression, and obstructive sleep apnea which was being treated with continuous positive airway pressure at night. Her home medications included inhaled albuterol on as-needed basis.

On examination, patient was noted to have distal extremity weakness and pronounced myotonia on shaking hands. Her height and weight were 170 cm and 63 kg, respectively. Heart rate (HR) was regular at 57 beats per minute. Blood pressure (BP) was 147/97 mmHg and respiratory rate was 18 per minute. The remainder of the physical exam was unremarkable.

Preoperative EKG showed sinus bradycardia. Transthoracic echocardiogram showed normal systolic function and no regional wall motion abnormalities. Due to her history of exertional dyspnea (on walking less than two blocks) associated with fatigue and exertional chest pain, her cardiologist decided to do cardiac nuclear stress test with lexiscan, which was negative for ischemia.

General anesthesia was chosen. The patient was brought into the warmed operating room with an 18-gauge (G) intravenous (IV) line in situ in the left arm. Monitors were applied to measure EKG, noninvasive BP, oxygen saturation, temperature, neuromuscular twitch, bispectral index (BIS), and urine output. Rapid sequence induction was accomplished by giving propofol (150 mg) and rocuronium (30 mg) intravenously. Intubation of the trachea with a 7.0 endotracheal tube was facilitated with a MAC 3 laryngoscope. Mechanical ventilation was initiated. A 20 G peripheral IV and 20 G radial arterial line were started in the right arm. Fentanyl 50 mcg was given after induction and propofol (120-150 mcg/kg/min) and remifentanil (0.2-0.3 mcg/kg/min) infusions were started. Warm intravenous fluids and a forced air warmer were used to maintain normothermia. Surgery lasted for three hours and the patient remained hemodynamically stable throughout. Surgeon infiltrated the incision sites with 0.25% bupivacaine. Neuromuscular monitoring, used on orbicularis oculi muscle, showed no twitches on train of four for the rest of the operation and no more muscle relaxant was given. Only at the end of the procedure, weak post-tetanic counts were noted without any train of four; hence sugammadex 240 mg IV was given for reversal of neuromuscular blockade. After a couple of minutes of giving sugammadex, patient started breathing spontaneously. Complete return of muscular strength was noted after ten minutes, approximately. Patient began to shiver while waking up and a forced air warmer was reapplied. Shivering subsided after a minute.

After making sure the patient was fully awake and breathing spontaneously, following commands with adequate muscle strength, the trachea was extubated. In the PACU, she received minimal IV narcotics (total of 35 mcg fentanyl) and IV acetaminophen (1 gm). Surgeon preferred to avoid nonsteroidal anti-inflammatory drugs (NSAIDs) in immediate postoperative period due to the risk of bleeding. She was kept warm with a forced air blanket and her vital signs remained stable. She was breathing comfortably with oxygen saturation above 98% on 2L/min.

The patient was observed in the recovery room for four hours during which time she was doing well without any signs or symptoms of respiratory distress. Her only complaint was about the brief shivering episode after waking up. Patient lived close to the hospital and had good family support; hence we decided to discharge her home and followed up with phone calls in the evening and the following morning. She was discharged home on oral NSAIDs as needed for pain control.

## 3. Discussion

Robotic assisted laparoscopic surgery with its extreme positioning and insufflation of the abdomen warrants general anesthesia. In patients with DM involving bulbar disease, rapid sequence induction is often chosen due to the high risk of aspiration. Maintenance of anesthesia with total intravenous anesthesia avoids volatile anesthetics which often cause shivering and hence precipitate myotonia. We used rocuronium as muscle relaxant as it is a nondepolarizing and intermediate acting agent and has been found to be safe in patients with DM as long as it is adequately reversed [1, 25]. The duration of muscle relaxation was longer than usual in our patient as found by no twitches on train of four and no posttetanic count on neuromuscular monitor throughout the operation. The longer duration could be attributed to increased sensitivity to muscle relaxant due to her underlying disease. Reversal with sugammadex was initially chosen over anticholinesterases as neostigmine has been found to cause incomplete reversal and depolarization in patients with DM [6, 11, 18, 26, 27, 30] but it was also indicated as our patient was in deep neuromuscular blockage at the end of the surgery as shown by the neuromuscular monitor. Sugammadex works by directly encapsulating, binding, and inactivating rocuronium. It has the ability to reverse any depth of neuromuscular blockage by rocuronium. Gurunathan et al had also described successful anesthetic management in patient with DM with general endotracheal, propofol and remifentanil infusion, and sugammadex for reversal like we did in our case [30].

We have reviewed the recent case reports for anesthesia in patients with DM in past 10 years using PubMed as our search engine and related citations.  Table 1 summarizes the cases with the type of surgery and type of anesthetics used. We have included cases which showed different anesthetic techniques used and if there were any untoward complications observed. Some cases have been described with regional anesthesia as primary technique. Gaszynski T. described a case of laparoscopic cholecystectomy which was successfully managed using dexmedetomidine (alpha-2 agonist) as infusion and without giving any opioid [8].

### 3.1. Myotonic Dystrophy

*Genetics of DM1*. DM is inherited in an autosomal dominant mode. There is a mutation in the DMPK (dystrophic myotonia protein kinase) gene on chromosome 19. The genetic lesion in DM1 (as in our case) is expansion of CTG trinucleotide repeat in untranslated region of DMPK [25, 31]. These repeats are transcribed into mRNAs which contains a CUG expansion and accumulates in ribonucleic inclusions in the nucleus. This leads to altered splicing of some genes like chloride channel receptor (CLC1), insulin receptor, and cardiac troponins. The altered synthesis of these genes is responsible for variable presentations in DM. There is mild correlation between repeat size and age of onset. Usually there are no symptoms if CTG repeat size is less than 100. Instability in the DNA increases with age and in subsequent generations. Larger repeat sizes are observed in muscle, heart, and brain tissues, which are most severely affected in DM. The chloride channel gene mutation results in decreased chloride conductance which is responsible for sustained depolarization manifesting as myotonia [25, 32].

### 3.2. Classic Symptoms of DM1

Depending on age of onset, DM1 has been divided into congenital, childhood onset and adult onset. Myotonia is not clinically presented before age of 3-5. The classic type of DM1 is the adult onset with first symptoms appearing between the ages of 15-35. It is characterized by myotonia, weakness of pharyngeal and neck muscles, muscles of mastication, distal limb muscles, ptosis, frontal baldness, and multisystem involvement including respiratory, cardiac, endocrine, and central nervous system [2]. The initial presentation is usually not muscle weakness, rather patients present with bilateral cataracts, arrhythmias, or infertility. DM 1 has broad range of clinical symptoms which are summarized below.

- *Muscle*: DM is characterized by myotonia and dystrophy of muscles. Myotonia is defined as persistent muscle contraction after termination of voluntary muscle contraction or stimulation. It was demonstrated in our patient during the hand grasp. It can also present in bulbar muscles resulting in difficulty in talking and swallowing. Myotonic contractures are painless and are usually not accompanied by weakness or are not related to changes in potassium concentrations, though shown to be made worse by exposure to cold [25]. Myotonia is not abolished by peripheral nerve block or neuromuscular junction blockage but infiltration with local anesthetics helps. Medications such as phenytoin, mexiletine, procainamide, and quinidine have been shown to be effective in abolishing myotonia [25]. Other muscular symptoms are distal muscle weakness and atrophy which might lead to foot drop, gait disturbances, and difficulty in performing fine tasks [31]. Disease may also involve muscles of face, larynx, and respiration. Typical facial features include ptosis, weak eyelid closure, and weak smile. Muscle weakness is progressive but slow and correlates with disease duration and CTG repeat expansion size [31].
- *Respiratory*: Upper airway and expiratory muscles are involved in early course and inspiratory muscles will be affected in severe disease. Expiratory muscle weakness leads to poor cough and atelectasis. Involvement of inspiratory muscles leads to poor inspiratory capacity and alveolar hypoventilation. Diaphragmatic muscles also become weak leading to higher risk of postoperative pulmonary complications (PPC) especially in upper abdominal surgery and older patients. Prevention of PPC requires active cleaning of upper airways, chest physiotherapy, and incentive spirometry [33, 34]. Weakness of pharyngeal muscles contributes to obstructive sleep apnea and aspiration pneumonia. Central ventilatory control mechanisms are abnormal leading to low central ventilatory drive and central sleep apnea [2]. Ventilatory response to carbon dioxide is reduced and chronic hypercapnia is a common finding.
- *Cardiac*: Conduction defects are common in patients with DM [31]. There is a progressive deterioration of the conducting system resulting in first degree heart block, bundle branch block, and widening of the QRS complex [25]. Sudden death has been associated with the development of third degree heart block. Less commonly, cardiomyopathy and congestive heart failure may occur. There is also an increased incidence of septal defects and valvular abnormalities, including mitral valve prolapse. Prophylactic insertion of pacemaker has been performed in patient with some conduction defects to prevent sudden cardiac death [1, 35].
- *Endocrine*: Hypothyroidism, primary gonadal failure, and abnormalities of glucose and insulin metabolism often with clinical diabetes mellitus can manifest in patients with DM [25].
- *Central nervous system*: Minor intellectual deficit, age related cognitive deficit, obsessive-compulsive, passive-aggressive behavior, and depression are common in patients with DM [1, 31]. Mental retardation is seen in 60% of patients with congenital DM.
- *Gastrointestinal*: Smooth muscle involvement may result in dysphagia, nausea, vomiting, reduction in rate of gastric emptying, and early satiety. Intestinal pseudoobstruction and esophageal aperistalsis have been reported in patients with DM [25, 31]. High incidence of gall stones is noted as well. There is higher risk of prolonged postoperative ileus, especially when opioids have been used.
- *Other features*: Patients may have other features as well such as frontal balding, cataracts, and testicular atrophy

*Pregnancy and DM*. Pregnancy might increase the muscle weakness and extramuscular manifestations. Increased risk of premature and prolonged labor, uterine atony, and postpartum hemorrhage has been reported [1].

*Course of Disease*. By the fifth or sixth decade, most patients become severely disabled due to recurrent chest infections due to aspirations and muscle weakness. Sudden cardiac death is known to cause death in one third of DM 1 patients [1]

### 3.3. Anesthesia and Myotonic Dystrophy

The first case report of anesthesia in DM was published in 1915 [16]. It was a muscle biopsy under ether general anesthesia [16, 36]. Operation was complicated by muscle spasm induced by surgical incision, leading to inadequate ventilation and cyanosis.

#### 3.3.1. Preoperative Period

Preoperative evaluation of patients with DM should involve multidisciplinary team including medical, neurology, cardiac, and anesthesiology specialties. Any preoperative weakness should be addressed and further evaluated. Mathieu et al. have defined a grading system which is useful in assessing the disease severity (Table 2). Our patient was found to be between grades 2 and 3 in MIRS grading. Twelve lead EKG is recommended because of high risk of conduction defects. Transthoracic echocardiogram should be considered if any cardiorespiratory symptom or abnormal EKG is reported. In patient with respiratory symptoms, consideration should be given to pulmonary function testing. Cardiac rhythm management device like pacemaker and defibrillators should be interrogated.

As patients are at higher risk of aspiration due to reduced gastric emptying and pharyngeal muscle dysfunction, preoperative administration of any sedatives should be avoided as it will also further add to the respiratory depression. Consideration should be given to preoperative prophylaxis with sodium citrate, metoclopramide, and H2 antagonists to prevent aspiration.

#### 3.3.2. Intraoperative Period

Choice of anesthesia should be regional wherever possible with minimal sedation. Spinal and epidural anesthesia have been reported to be successful in the past by Corriea et al., Subramaniyam et al., and Uno et al. [6, 7, 9], either as a sole anesthetic or as part of postoperative analgesia. As there are case reports of shivering and precipitation of myotonic crisis with uterine atony after epidural anesthesia for Cesarean section [21, 37], patients should be closely monitored during spinal or epidural anesthesia. When general anesthesia is chosen, anesthesiologist should be extremely careful during all the phases. During general anesthesia, muscle relaxants should be avoided as much as possible especially if surgery does not require it. If muscle relaxants need to be given, it should be ensured that patient is completely reversed. Use of peripheral nerve blocks in DM has seldom been reported because of the fear that neurostimulation during the peripheral nerve block might precipitate myotonia. But it may not be an issue these days as nerve stimulator can be avoided due to ultrasound availability [1].

Patients are very sensitive to opiates and anesthetic agents [3].  Table 3 summarizes the anesthetic drugs and their use in patients with myotonic dystrophy. Apart from standard ASA monitors, neuromuscular block monitor and temperature monitors should be applied. Rapid sequence induction should be the chosen mode of induction as these patients are at higher risk of aspiration due to pharyngeal muscle weakness.

DM can be precipitated intraoperatively by hypothermia, shivering, surgical or mechanical stimulation, and electrocautery [22, 25, 38]. It is important to maintain normothermia using forced air blankets and fluid warmers and maintaining operating room temperature. Availability of temporary pacemaker and defibrillator should be made ensured as well.

#### 3.3.3. Postoperative Period

Numerous cases of perioperative complications have been reported in patients with DM and most of them are pulmonary [34, 38]. Hence, careful monitoring is required in the early postoperative period [34]. Postoperative observation for 24 hours was suggested in the past for patients with DM receiving general anesthesia [39]. It is recommended to avoid use of opioids in recovery room if possible and consider multimodal treatment for pain.

In a retrospective study of 219 DM patients in Quebec between periods of 1961 and 1986, Mathieu J. et al. found the rate of perioperative complications to be 8.2%, which were mostly pulmonary. Major risk factors for them were found to be upper abdominal surgery and severe muscular disability as assessed by proximal muscle weakness. The likelihood was not related to any specific anesthetic drugs [34].

In 2009, Sinclair JL et al. reported a retrospective study on 27 pediatric patients, who received anesthesia 78 times over a span of 18 years (1989-2006) [40]. Overall postoperative pulmonary complication was 10%. Significant risk factors identified were high MIRS score, use of muscle relaxants without reversal, use of perioperative morphine, longer duration of surgery (>1 hour), and increased CTG repeat size. Four intraoperative and 8 postoperative adverse events were recorded, which were mostly pulmonary like hypoxia and apnea intraoperatively and hypoventilation, failed weaning, cyanosis, and continued mechanical ventilation in ICU postoperatively. [40]. Use of muscle relaxants without reversal was reported to be one of the major factors for postoperative complications. But in the cases after year of 2000, there was less use of muscle relaxants altogether, so practitioners were avoiding both muscle relaxants and reversal. In recent past, sugammadex has been used with great success for these cases, avoiding the use of anticholinesterases and successfully reversing intermediate acting steroidal muscle relaxants. In a retrospective review of 121 patients with DM2 by Kirzinger et al., authors found the complications to be much less in DM2 patients as compared to DM1 patients. It was mainly due to minor respiratory involvement in DM2 patients. Risk factors for increased perioperative complications are summarized in Table 4 [18, 22, 23, 34, 40].

We decided to discharge our patient after making sure she is doing well in the recovery room because of her minimal invasive lower abdominal surgery, low to moderate MIRS score, good family support, and nearby residence. We followed up with the patient on phone and ensured her well-being.  Table 5 summarizes the management for anesthesia in patient with myotonic dystrophy.

## 4. Limitations

We attempted to comprehensively review evidence-based anesthetic management of patients with this rare disease; however we found most of recommendations in the literature based upon case reports and retrospective reviews rather than prospective randomized controlled trials. In our case, we could have used regional anesthesia such as transversus abdominis plane block or epidural anesthesia for postoperative pain control; however patient did not consent for that.

## 5. Conclusion

Myotonic dystrophy is a rare multisystemic disease and is not commonly encountered in day to day clinical practice. With careful preoperative assessment and evaluation of the patient, anesthesia plan can be formulated and many intra- and postoperative complications can be avoided. Although retrospective studies had recommended observation of the patient overnight after general anesthesia, in our experience it can be decided on case to case basis depending on disease severity, type of surgery, and other risk factors regarding the discharge to minimize hospital admissions.

## Figures and Tables

**Table 1:** Review of reported cases of anesthesia in patients with myotonic dystrophy over last ten years.

Author	Type of Anesthesia	Type of Surgery	Complications

Gorelick L. et al. 2018 Case series (3 cases) [5]	MAC sedation and general	EP study and pacemaker insertion	One patient develops arrhythmias/atrial fibrillation during the procedure

Uno et al. 2017 [6]	General with propofol infusion, rocuronium, and sugammadex for reversal. TAP block and epidural for pain	Laparoscopic cholecystectomy	none

Subramaniam et al. 2016 [7]	General and epidural	Open resection of pheochromocytoma/paraganglioma	none

Gaszynski T. 2016 [8]	General with propofol and dexmedetomidine infusions	laparoscopic cholecystectomy	none

Corriea M. et al. 2016 [9]	Continuous spinal	Laparoscopic cholecystectomy	none

Gurunathan U. et al. 2015 [8]	General with propofol/remifentanil infusion and reversal with sugammadex	Laparoscopic cholecystectomy	none

Urciuoli P. et al. 2014 Case series (2 cases) [10]	General and thoracic epidural anesthesia without muscle relaxant	Laparoscopic cholecystectomy	none

Piccard A. et al. 2013[11]	General and sugammadex. use of acceleromyography	PEG insertion, orchiopexy, and tongue tie release	congenital MD

Stourac P. et al. 2013 [12] Case series(2 cases)	General with sugammadex reversal	Cesarean section x 2 in same patient	remain intubated in ICU for 2 hours first time

Bissinoto FM et al.[13]	General with propofol and remifentanil infusion and reversal with prostigmine	Laparoscopic cholecystectomy	Patient developed myotonia after extubation, unable to intubate, maintained ventilation with LMA for few hours.

**Table 2:** Muscular impairment rating scale [1].

Grade	Description
Grade 1	No muscular impairment

Grade 2	Minimal signs: myotonia, facial weakness, jaw and temporal wasting, ptosis, no distal weakness except isolated digit flexor weakness

Grade 3	Distal weakness, no proximal weakness except isolated elbow extensor weakness

Grade 4	Mild to moderate proximal weakness

Grade 5	Severe proximal weakness

**Table 3:** Anesthetic drugs and use in myotonic dystrophy patients.

Drug	Use in DM	Adverse effects/complications	Other effects
Propofol	Demonstrated to be safe in many reported cases [2, 14, 15]	Can induce myotonia [16, 17] Prolonged recovery after targeted controlled infusions. [11]	Sensitivity is altered. Less than 1mg/kg has been shown to be enough to induce general anesthesia and intubation by Speedy et al. [16]

Thiopentone	Has been used in multiple case reports/series without any adverse event [18].	Shown to result in unexpected apnea/prolonged respiratory depression [3, 13]	Prolonged apnea due to thiopentone seems to be idiosyncratic.

Etomidate	Use has been described before [19]	Continuous infusion not recommended due to steroid suppression [2, 20].	

Volatile Anesthetics	Has been reported to be used with success [21]	Concern with deep inhalation anesthesia leading to shivering and compromised cardiac status	Some reluctance to use initially due to association with malignant hyperthermia (MH). But recent literature showed DM patients are no more susceptible to MH than general population. [22, 23]

Muscle relaxants	Shorter and intermediate acting nondepolarizing agents are safe to be used. Atracurium has been described to be used without reversal agent or prolonged muscle weakness [24].	Depolarizing muscle relaxants has been shown to induce myotonia leading to inability to ventilate and maintain the airway [3, 25]. Longer acting nondepolarizing agents have been shown to add to the weakness/respiratory depression. [25].	Avoidance of muscle relaxants, if possible, is recommended.

Reversal agents	Sugammadex has been reported to be safe and has been used multiple times in recent past [26].	Incomplete reversal and postoperative breathing difficulties have been noted in multiple case reports where neostigmine was used [18]	Avoid use of anticholinesterases for reversal [27], exact mechanism not known.

Dexmedetomidine	Successful use of dexmedetomidine also has been reported to provide opioid free anesthesia in many recent case reports [8]	none reported	

Opioids	Use of shorter acting opioids is recommended for intraoperative period.	Higher risk of respiratory depression. Higher risk of postoperative ileus.	Avoid opioids in preoperative and postoperative period.

Total intravenous anesthesia	Use of propofol and remifentanil infusion have been reported before in cases of DM [13, 28]	Remifentanil has been linked to postoperative hyperalgesia [29].	

**Table 4:** Risk factors for perioperative complications [13, 30].

Upper abdominal surgery
Longer duration of surgery (>1 hour)
Severe muscular disability
High MIRS score
Use of muscle relaxants without reversal
Use of perioperative morphine
Increased CTG repeat size

**Table 5:** Summary of anesthesia management in patient with myotonic dystrophy.

Preoperative	Assess the type and duration of surgery. If it is emergent or upper abdominal surgery, expected duration longer than 1 hour, have posted bed available in ICU, patient may require post-op mechanical ventilation Assess patients muscle weakness by using MIRS score or Gilliam's grading. If it is of higher grade, careful postoperative monitoring is required. Check patient's glucose levels Twelve lead EKG and echocardiography because of higher risk of conduction defects. Consider pulmonary function testing if there are cardiorespiratory symptoms. Interrogation of cardiac rhythm management devices, if any. Avoid premedication with opioids. Consider antiaspiration prophylaxis with sodium citrate, H2 antagonist, metoclopramide.

Intraoperative	Choice of anesthesia local or regional if possible If general, avoid muscle relaxants if the surgery does not require them. If muscle relaxants are given, consider reversal. Maintain normothermia using thermal blankets, fluid warmers, and higher operating room temperature. Avoid shivering Invasive blood pressure monitoring, neuromuscular block monitoring, and temperature monitoring along with standard ASA monitors. Rapid sequence induction because of pharyngeal weakness and possibility of aspiration. Avoid drugs like Succinylcholine and anticholinesterases like Neostigmine. Have pacemaker/defibrillator in room if patient has conduction abnormalities. Consider Sugammadex for reversal Regional for postoperative pain control.

Postoperative	Continuous pulse oximetry and EKG monitoring for prolonged period. Consider postoperative ventilation in patient with high risk factors of developing pulmonary complications. Restrict use of opioids to minimal Consider multimodal pain management. Aggressive pulmonary toilet with incentive spirometry, chest physiotherapy, and cough assistant devices.

## Abbreviations

DM: Myotonic dystrophy
OFA: Opioid-free anesthesia
EKG: Electrocardiogram
PPC: Postoperative pulmonary complications
MIRS: Muscular impairment rating scale
NSAIDs: Nonsteroidal anti-inflammatory drugs
ICU: Intensive care unit.

## References

[1] Veyckemans F., Scholtes J.-L. (2013). Myotonic dystrophies type 1 and 2: anesthetic care. *Pediatric Anesthesia*.

[2] Bennun M., Goldstein B., Finkelstein Y., Jedeikin R. (2000). Continuous propofol anaesthesia for patients with myotonic dystrophy. *British Journal of Anaesthesia*.

[3] Mitchell M. M., Ali H. H., Savarese J. J. (1978). Myotonia and neuromuscular blocking agents. *Anesthesiology*.

[4] Kirzinger L., Schmidt A., Kornblum C., Schneider-Gold C., Kress W., Schoser B. (2010). Side effects of anesthesia in DM2 as compared to DM1: a comparative retrospective study. *European Journal of Neurology*.

[5] Gorelik L., Flores A. (2018). Anesthetic management for multiple family members with myotonic dystrophy for interventional cardiac procedures—a case series. *Frontiers in Medicine*.

[6] Uno R., Matsuda S., Murao K. (2017). Use of sugammadex in a patient with myotonic dystrophy undergoing laparoscopic cholecystectomy. *The Japanese journal of anesthesiology*.

[7] Subramaniam A., Grauer R., Beilby D., Tiruvoipati R. (2016). Anesthetic management of a myotonic dystrophy patient with paraganglionoma. *Journal of Clinical Anesthesia*.

[8] Gaszynski T. (2016). Opioid-free general anesthesia in patient with Steinert syndrome (myotonic dystrophy): case report. *Medicine (United States)*.

[9] Correia M., dos Santos A., Lages N., Correia C. (2016). Laparoscopic cholecystectomy under continuous spinal anesthesia in a patient with Steinert's disease. *Brazilian Journal of Anesthesiology*.

[10] Urciuoli P., Buonopane C. E., Sottile D. (2014). Laparoscopic cholecystectomy in steinert's myotonic dystrophy: About two clinical cases. *Annali Italiani Di Chirurgia*.

[11] Pickard A., Lobo C., Stoddart P. A. (2013). The effect of rocuronium and sugammadex on neuromuscular blockade in a child with congenital myotonic dystrophy type 1. *Pediatric Anesthesia*.

[12] Stourac P., Krikava I., Seidlova J. (2013). Sugammadex in a parturient with myotonic dystrophy. *British Journal of Anaesthesia*.

[13] Bisinotto F. M. B., Fabri D. C., Calçado M. S., Perfeito P. B., Tostes L. V., Sousa G. D. (2010). Anesthesia for videolaparoscopic cholecystectomy in a patient with Steinert disease. case report and review of the literature. *Revista Brasileira de Anestesiologia*.

[14] White D. A., Smyth D. G. (1989). Continuous infusion of propofol in dystrophia myotonica. *Canadian Journal of Anesthesia*.

[15] Milligan K. A. (1988). Propofol and dystrophia myotonica. *Anaesthesia*.

[16] Speedy H. (1990). Exaggerated physiological responses to propofol in myotonic dystrophy. *British Journal of Anaesthesia*.

[17] Bouly A., Nathan N., Feiss P. (1991). Propofol in myotonic dystrophy. *Anaesthesia*.

[18] Aldridge L. M. (1985). Anaesthetic problems in myotonic dystrophy: a case report and review of the aberdeen experience comprising 48 general anaesthetics in a further 16 patients. *British Journal of Anaesthesia*.

[19] Müller H., Puntvan Manen J. A. (1982). Maxillo-facial deformities in patients with dystrophia myotonica and the anaesthetic implications. *Journal of Maxillofacial Surgery*.

[20] Wagner R. L., White P. F., Kan P. B., Rosenthal M. H., Feldman D. (1984). Inhibition of adrenal steroidogenesis by the anesthetic etomidate. *The New England Journal of Medicine*.

[21] Paterson R. A., Tousignant M., Skene D. S. (1985). Caesarean section for twins in a patient with myotonic dystrophy. *Canadian Anaesthetists' Society Journal*.

[22] Gandhi R., Jain A. K., Sood J. (2011). Desflurane anaesthesia in myotonic dystrophy. *Indian Journal of Anaesthesia*.

[23] Parness J., Bandschapp O., Girard T. (2009). The myotonias and susceptibility to malignant hyperthermia. *Anesthesia & Analgesia*.

[24] Nightingale P., Healy T. E. J., Mcguinness K. (1985). Dystrophia myotonica and atracurium: a case report. *British Journal of Anaesthesia*.

[25] Russell S. H., Hirsch N. P. (1994). Anaesthesia and myotonia. *British Journal of Anaesthesia*.

[26] Matsuki Y., Hirose M., Tabata M., Nobukawa Y., Shigemi K. (2011). The use of sugammadex in a patient with myotonic dystrophy. *European Journal of Anaesthesiology*.

[27] Buzello W., Krieg N., Schuckewei A. (1982). Hazards of neostigmine in patients with neuromuscular disorders. *British Journal of Anaesthesia*.

[28] Nakanishi T., Nishihama M., Hirai A., Uchikado M., Shimosaka M., Uehara A. (2010). Successful management of a patient with myotonic dystrophy under total intravenous anesthesia with propofol, remifentanil and rocuronium bromide, combined with epidural anesthesia. *The Japanese Journal of Anesthesiology*.

[29] Weinbroum A. A. (2015). Role of anaesthetics and opioids in perioperative hyperalgesia. *European Journal of Anaesthesiology*.

[30] Gurunathan U., Duncan G. (2015). Successful use of sugammadex and uneventful recovery from general anaesthesia in a patient with myotonic dystrophy. *Indian Journal of Anaesthesia*.

[31] Bird T. D. (1999). Myotonic dystrophy type 1. *GeneReviews®*.

[32] Franke C., Hatt H., Iaizzo P. A., Lehmann‐Horn F. (1990). Characteristics of Na+ channels and Cl‐ conductance in resealed muscle fibre segments from patients with myotonic dystrophy. *The Journal of Physiology*.

[33] Bogaard J. M., van der Meche F. G. A., Hendriks I., Ververs C. (1992). Pulmonary function and resting breathing pattern in myotonic dystrophy. *Lung: An International Journal on Lungs, Airways and Breathing*.

[34] Mathieu J., Allard P., Gobeil G., Girard M., De Braekeleer M., Bégin P. (1997). Anesthetic and surgical complications in 219 cases of myotonic dystrophy. *Neurology*.

[35] Lazarus A., Varin J., Babuty D., Anselme F. R., Coste J., Duboc D. (2002). Long-term follow-up of arrhythmias in patients with myotonic dystrophy treated by pacing: a multicenter diagnostic pacemaker study. *Journal of the American College of Cardiology*.

[36] Johnson W., Marshall G. (1915). Observations on thomsen's disease. *QJM: An International Journal of Medicine*.

[37] Harris M. N. (1984). Extradurnl analgesia and dystrophia myotonia. *Anaesthesia*.

[38] Mudge B. J., Taylor P. B., Vanderspek A. F. L. (1980). Perioperative hazards in myotonic dystrophy. *Anaesthesia*.

[39] Cox M., Lawton G., Gowrie-Mohan S. (1995). Ambulatory extradural analgesia. *British Journal of Anaesthesia*.

[40] Sinclair J. L., Reed P. W. (2009). Risk factors for perioperative adverse events in children with myotonic dystrophy. *Pediatric Anesthesia*.

